# Low-Frequency Vibrational Density of States of Nanophase-Separated Poly(*n*‑alkyl methacrylate)s: Confined Phonons and Relationship to Specific Heat

**DOI:** 10.1021/acs.macromol.5c00898

**Published:** 2025-07-09

**Authors:** Paulina Szymoniak, Fanni Juranyi, Margarita Kruteva, Reiner Zorn, Andreas Schönhals

**Affiliations:** 1 42220 Bundesanstalt für Materialforschung und-prüfung (BAM), Unter den Eichen 87, Berlin 12205, Germany; 2 PSI Center for Neutron and Muon Sciences, Villigen PSI 5232, Switzerland; 3 28334 Forschungszentrum Jülich GmbH, Jülich Centre for Neutron Science (JCNS-1), Jülich 52425, Germany; 4 Institut für Chemie, Technische Universität Berlin, Straße des 17. Juni 135, Berlin 10623, Germany

## Abstract

This study investigates the low-frequency vibrational density of states of nanophase-separated poly­(*n*-alkyl methacrylate)­s (PnMAs) and its relationship to specific heat. This system undergoes a nanophase separation for *n* > 1 in alkyl side chain-rich domains and a backbone-rich matrix. Using inelastic neutron scattering, the low-frequency vibrational density of states (Boson peak, BP) of PnMAs with varying alkyl side chain lengths (methyl, butyl, hexyl, and octyl) is measured. The results reveal that the BP shifts to higher frequencies with increasing side chain length reaching a maximum. This result indicates a counterbalance of confinement effects and the scattering of the matrix. The behavior of the Boson peak of the PnMAs is compared to other nanophase-separated systems, such as Janus-polynorbornenes and hexakis­(*n*-alkyloxy)­triphenylene discotic liquid crystals. The study also explores the connection between the BP and specific heat capacity, showing a linear relationship between the maximum frequency of the BP and the maximum temperature of the specific heat capacity anomaly. Moreover, from the measured vibrational density of states, the specific heat capacity is calculated and compared to experimental results. The comparison reveals qualitative agreement between the calculated and experimental data. These findings provide evidence for the sound wave interpretation of the Boson peak.

## Graphical abstract

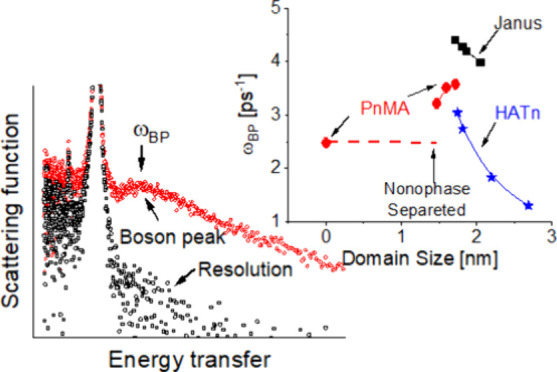

## 1 Introduction

Glasses, including polymer glasses, show characteristic excess contributions to the low-frequency vibrational density of states (VDOS). These excess contributions appear approximately in the frequency range from 1.5 to 7.5 ps^–1^, which corresponds to energies of 1–5 meV. For crystalline materials, the Debye model of sound waves predicts for the VDOS *g*(ω) ∼ ω^2^ where ω denotes the angular frequency. In the reduced representation *g*(ω)/ω^2^ versus ω, the excess contributions observed for glasses show up as a peak, which is called the Boson peak (BP).^1^ The vibrations related to the BP are discussed to be also responsible for the anomaly of the temperature dependence of the specific heat at low temperatures. It also does not follow the prediction of the Einstein/Debye model *c_p_* ∼ *T* ^3^ found to be valid for crystalline solids. Plotting *c_p_*/*T* ^3^ versus temperature, a peak is observed like for the BP. The maximum temperature $T_{\max,c_{p}}$ in the specific heat representation is related to the maximum frequency of the Boson peak ω_BP_ by

$$k_{B}T_{\max,c_{p}}=\hbar \omega_{\mathrm{BP}}/A$$ 1

*k*_B_ is the Boltzmann constant; ℏ is Planck's constant divided by 2π. It is worth noting that the exact proportionality factor *A* in eq 1 between $T_{\max,c_{p}}$ and ω_BP_ depends slightly on the shape of the Boson peak.

As discussed, the BP appears in the frequency range of ps^–1^ while the process of glass formation from a liquid or melt (glass transition) at the glass transition temperature *T*_g_ takes place at timescales that are by orders of magnitude longer, in the range of 100 s or even longer. However, it is discussed that the Boson peak is related to the glass transition.^2,3^ There is some agreement in the literature that the glass transition is due to cooperative effects.^4–6^ The cooperativity approach to the glass transition was pioneered by Adam and Gibbs introducing the cooperatively rearranging regions (CRR).^7^ A CRR is defined as the smallest region that can change its configuration independently from its neighbors. Wynne et al. assigned the Boson peak to clusters consisting of 20 molecules,^8^ which might be correlated with CRR. Some further evidence was provided by simulations for symmetrical molecules. In that approach, the appearance of the BP was related to the presence of structural and dynamic heterogeneities, which can be also discussed within the cooperativity approach.^9^

Most researchers agree that the BP is a universal property of glasses or more general of amorphous materials. Nevertheless, a Boson peak is also observed for materials that have a partial order like plastic crystals.^10–13^

The molecular origin of the Boson peak is still under debate. The existing theories concerning the BP can be categorized roughly into three groups. One theory is known as a soft potential approach (see for instance refs 14–16 ). Here, it is assumed that the Boson peak results from quasi-localized vibrations. Groups of atoms are subject to a soft potential related to the peculiarities of the interatomic forces due to the amorphous structure of the material under study. It is important to note that the vibrations under consideration in the soft potential are fundamentally different from the propagation of sound waves. In the second theoretical approach to the Boson peak, a hypothetical crystalline structure is assumed as the edge limit of the amorphous state. In this context, the Boson peak is understood as a broadened and frequency-shifted manifestation of the Van Hove singularity observed in crystalline systems where the broadening and frequency shift arise from the amorphous structure.^17^ It is worth nothing that ref 11 posits that an interaction between the optical and acoustical dispersion branches can result in the emergence of a pseudo Van Hove singularity. Schirmacher et al.^18^ proposed a third approach that integrates elements from the two previously discussed categories. In this framework, the Boson peak is attributed to harmonic vibrations occurring within a disordered environment, which retain characteristics of sound waves. This approach assumes that the Boson peak is not a modification of the Van Hove singularity, and the introduced “soft spots” are analogous to the soft potential model.^19^ In the latter discussed two theoretical frameworks, the Boson peak in amorphous systems is thought to originate from sound waves, with the vibrational density of states of the corresponding hypothetical ordered system being altered by the amorphous disordered structure. Within the framework of the sound wave model, the maximum frequency of the Boson peak is associated with the transverse sound velocity *c*_t_ and a characteristic length scale (see, for example, refs 20–22 ) by

$$\xi ={Bc}_{t}/\omega_{\mathrm{BP}}$$ 2

In eq 2, ω_BP_ is the maximum frequency of the Boson peak, ξ is a characteristic length scale, and *B* denotes a constant in the order of one. The transverse sound velocity is related to the elastic modulus. An increase in the elastic modulus is associated with a corresponding increase in *c*_t_, whereas a decrease in the modulus leads to a reduction in *c*_t_. It is further noteworthy that deviations from eq 2 have been observed in certain highly specialized systems.^23^

Besides the approaches discussed briefly above, employing numerical simulation the BP can also be related to a local breaking of center-inversion symmetry.^24^

The Boson peak has been studied in low-molecular-weight glass-forming materials and polymers, including those in nanoscale confinement. These experimental data suggest that the Boson peak in amorphous materials can be attributed to collective phenomena such as sound waves.^25–29^ Such an interpretation of the Boson peak was also supported by simulation (see for example refs 30 and 31 and references cited). Furthermore, the sound wave approach to the BP was also in agreement with inelastic neutron scattering investigations of the VDOS of polymers of intrinsic microporosity (PIM-1 and PIM-EA-TB),^32,33^ microporous Si-substituted polynorbornenes,^34^ and polynorbornenes with bulky carbocyclic side groups.^35^

Recently, the vibrational low-frequency density of states was investigated for a homologous series of addition-polymerized “Janus” poly­(tricyclononenes) (called Janus-polynorbornenes here) employing also inelastic neutron scattering.^36^ These macromolecules are composed of a semirigid polynorbornene backbone, with three flexible alkyl side chains per repeating unit, where the length of the side chains is systematically varied.^37,38^ A comprehensive study of the molecular mobility of these polymers was further conducted elsewhere.^39^ One key finding was that these polymers exhibit nanophase separation into alkyl side chain-rich domains distributed in a backbone-rich matrix. The size of the alkyl side chain-rich domains, as determined by X-ray scattering, increases with the length of the alkyl side chains. The glass transition of the alkyl side chain-rich domains was identified as a hindered glass transition,^39,40^ which was also supported by a quasielastic neutron scattering study for this system.^41^ For the VDOS of these polymers, it was observed that the Boson peak is located at higher frequencies compared to conventional polymers like poly­(phenyl methyl siloxane).^25,42^ With increasing size of the of the nanophase-separated alkyl side chain-rich domains, the maximum frequency of the Boson peak shifts to lower frequencies, which could be understood by confined phonons.

A corresponding behavior of the low-frequency vibrational density was observed for a homologous series of hexakis­(*n*-alkyloxy)­triphenylene (HATn).^10^ In the condensed state, the HATn materials can form a discotic hexagonal ordered liquid crystal besides a plastic crystal at lower temperatures. In this structure, the triphenylene cores of the HATn molecules self-assemble into one-dimensional columns, which arrange in a hexagonal lattice. The six alkyl side chains per building molecule fill the intercolumnar space yielding also a nanophase-separated morphology. A related structure can be expected for the plastic crystalline state. It is worth noting that both the intercolumnar space filled with the alkyl chains as well as the self-assembled columns undergo separate glass transitions.^43,44^ By inelastic neutron scattering, it was revealed that the Boson peak found for these materials shifts like for the Janus-polynorbornenes to lower frequencies with increasing length of the alkyl side chains, which could be also understood by confined phonons.

It was first shown by Beiner and Huth that conventional poly­(*n*-alkyl methacrylate)­s also undergo a nanophase separation into alkyl side chain-rich domains distributed into a backbone-rich matrix.^40^ This nanophase separation is confirmed by X-ray measurements as well as by the observation of two different glassy dynamics related to different glass transitions where that of alkyl side chain-rich domains is characterized as a hindered glass transition.^40^ An investigation of the low-temperature dependence of the heat capacity shows characteristic anomalies, which are argued to be due to excess contributions to the low-frequency vibrational density.^2^ Therefore, a study of the Boson peak in poly­(*n*-alkyl methacrylates) is crucial for an the understanding of the underlying mechanisms that govern the low-frequency vibrational density of states in these nanophase-separated materials. This research could contribute to a more comprehensive understanding of the correct theoretical framework and the molecular origins of the Boson peak. In this work a detailed discussion of the dependence of the Boson peak on the length of the alkyl side chain and the characteristic size of the alkyl side chain-rich domains is presented. The obtained results are also interrelated to the data of the Janus-polynorbornenes and HATn discotic liquid crystals. The connection of the Boson peak to the specific heat capacity is discussed in detail. It is worth noting that the molecular mobility of the poly­(*n*-alkyl methacrylate)­s was studied elsewhere.^45–48^

## 2 Experimental Section

### 2.1 Materials

The general structure of the poly­(*n*-alkyl methacrylate)­s (PnMA) is given in Figure 1. PnMA samples with side chain length methyl (*n* = 1), butyl (*n* = 4), hexyl (*n* = 6), and octyl (*n* = 8) were purchased from Polymer Source (Montreal, Canada). Some details of the samples are given in Table 1. The samples were used without further purification.

**Figure 1:**
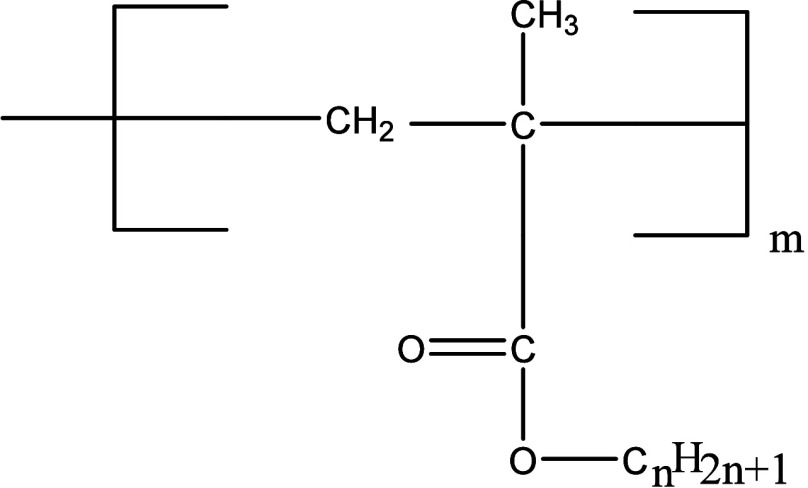
Chemical structure of the poly­(*n*-alkyl methacrylate)­s. *n* is the number of the carbon atoms in the alkyl side chain, whereas *m* denotes the degree of polymerization.

**Table 1:** Molecular Details of the Investigated Samples^a^

name	code	*M*_n_ 10^3^ [g/mol]	PDI	*T*_g_ [K]	domain size [nm]
poly(methyl methacrylate)	P1MA	42.9	1.04	404	
poly(butyl methacrylate)	P4MA	38.0	1.15	292	1.47
poly(hexyl methacrylate)	P6MA	30.0	1.08	270	1.59
poly(octyl methacrylate)	P8MA	43	1.02	251	1.72

a The molecular weights were provided by the producer. The glass transition temperatures *T*_g_ were measured with a PerkinElmer DSC 8500 at a heating rate of 10 K/min (second heating run). The sizes of the alkyl side chain-rich domains were taken from ref 40, estimated by a simple Bragg equation as the shape of the domains are not known. Please note that a nanophase separation is only observed for *n* > 1.^40^ The dependence of the domain size on the number of the carbon atoms is given in the Supporting Information (Figure S1).

From the obtained powder samples, films were prepared by hot pressing at temperatures *T* = *T*_g_ + 55–75 K employing a sample press exerting a pressure of 10–90 kN on a film with a final area of about 100 cm^2^. The pressing conditions were selected according to the polymer and its degradation temperature. The thickness of the samples was adjusted to ensure for ca. 10% neutron scattering. Then, the sample films were inserted in flat aluminum containers as Al is nearly transparent for neutrons.

It was shown in ref 40 that for longer alkyl chains (*n* > 1), the PnMA materials undergo a nanophase separation into alkyl side chain-rich domains surrounded by a backbone-rich matrix. The literature lacks a consensus on whether the morphology of polymers with long *n*-alkyl side chains is quasi-one-dimensional (interdigitated) or three-dimensional.^40^

### 2.2 Neutron Scattering

The cold neutron time-of-flight spectrometer FOCUS, located at SINQ at the Paul Scherrer Institute in Villigen, Switzerland, was employed for the inelastic neutron scattering experiment. The incident neutron beam had a wavelength of 5 Å. For the measurement of the vibrational density of states (VDOS), the full width of the 29 mm wide sample, oriented at a 30° angle relative to the incoming beam, was utilized. This configuration yielded an energy resolution (full width at half-maximum, fwhm) ranging from 80 to 125 μeV, contingent on the scattering angle. The energy resolution was determined by measuring the sample at a temperature of 2 K, under the assumption that all molecular fluctuations and vibrations were immobilized. The VDOS was measured at 50 K to minimize the impact of quasielastic scattering on the recorded signal. The sample temperature was controlled using a helium cryostat.

Data reduction was performed using DAVE software.^49^ For the calculation of the VDOS, detectors were grouped by angle into 20 groups spanning angles from 14 to 124°, corresponding to elastic scattering vectors of 0.3 to 2.2 Å^–1^. A custom-made Python script was employed to correct the data for self-shielding effects in a plate. An example for the raw data measured at FOCUS is given in Figure 2.

**Figure 2:**
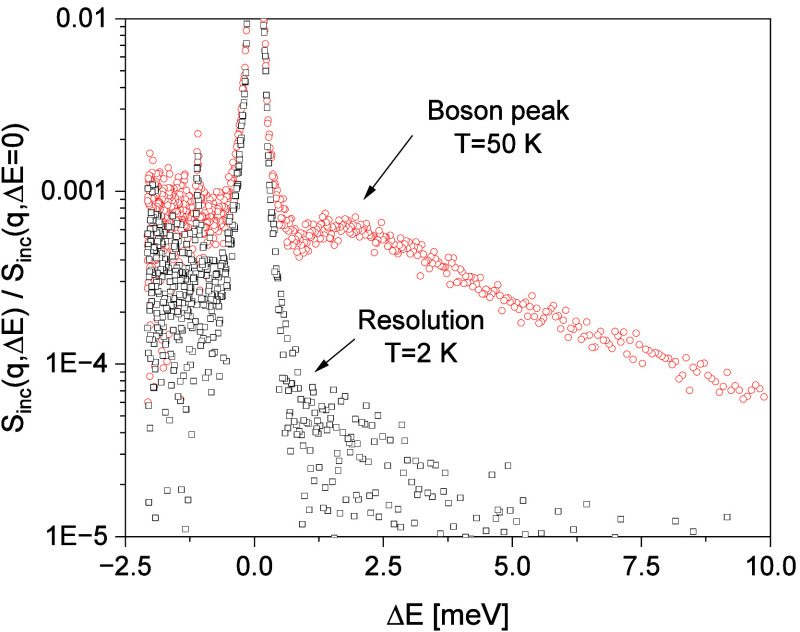
FOCUS spectra for P1MA at a scattering vector of 2.2 Å^–1^ (angle 124°) normalized to the height of the elastic line: Black squares: resolution of FOCUS measured at 2 K. Red circles: inelastic scattering measured at 50 K. Δ*E* is the measured energy transfer, see below.

## 3 Results and Discussion

In a neutron scattering experiment, energy and momentum are transferred between neutrons and the nuclei of the material under investigation. This interaction provides insights into the spatial and temporal characteristics of the process being studied.^50^ The primary experimental quantity measured in neutron spectroscopy is the double differential cross section, which is defined as

$$\frac{d^{2}\sigma }{d\Omega d\omega }=\frac{1}{4\pi }\frac{k_{f}}{k_{i}}(\sigma_{\mathrm{coh}}S_{\mathrm{coh}}(q,\omega )+\sigma_{\mathrm{inc}}S_{\mathrm{inc}}(q,\omega ))$$ 3

In the neutron scattering experiments, the incident and final wave vectors of the neutron beam are denoted by ***k*_i_** and ***k*_f_**. The difference between these vectors defines the scattering vector **q**. In the following, because of the isotropy of the materials, only the modulus *q* of **q** is considered. The coherent and incoherent dynamic structure factors are represented by *S*_coh_(*q*,ω) and *S*_inc_(*q*,ω), respectively. The measured energy transfer Δ*E* is related to the angular frequency ω by ω = Δ*E*/ℏ. σ_coh_ and σ_inc_ are the scattering cross sections for coherent and incoherent scattering, respectively. All PnMA polymers contain hydrogen (H), carbon (C), and oxygen (O) nuclei (see Figure 1). The corresponding scattering cross sections calculated for the repeating unit of the polymers are provided in Table 2, with detailed calculations available in the Supporting Information (see Table S1).

**Table 2:** Scattering Cross Sections for Incoherent and Coherent Scattering of the Repeating Units of the PnMA Materials

monomeric unit	σ_coh_ [mm^–1^]	σ_inc_ [mm^–1^]	scattering contribution of the alkyl side chains [%]
P1MA	0.0366	0.5076	32.5
P4MA	0.0356	0.5417	59.3
P6MA	0.0345	0.54	67.8
P8MA	0.0339	0.5416	73.3

The calculated cross sections for the polymers indicate that the experimentally observed scattering is predominantly incoherent, primarily due to the high incoherent scattering cross section of the hydrogen nuclei. These hydrogen nuclei are mostly located in the alkyl side chains. Consequently, the nanophase-separated structure of the PnMA for *n* > 1 results in the major contribution of the scattering originating from the alkyl side chain-rich domains, which increases with increasing length of the side chain (see Table 2).

The method for estimating the density of states from scattering data relies on the standard expression for one-phonon scattering within the incoherent approximation, applicable to a single type of nucleus.^51^ In this approach, the density of states is connected to the incoherent scattering structure factor by

$$S_{\mathrm{inc}}(q,\omega )=e^{-2W(q)}(\delta (\omega )+\frac{\hbar q^{2}}{2\mathrm{m̅}}\frac{g(\omega )}{\omega }\times (\exp(\frac{\hbar \omega }{k_{B}T})-1{)}^{-1})$$ 4

In eq 4, the Debye–Waller factor is represented by the term e^–2*W*(*q*)^ and δ is the Dirac or delta function. The average atomic mass is denoted by *m̅*. The dynamics of hydrogen are disproportionately emphasized in the results due to its high incoherent scattering cross section and low mass. However, if hydrogen nuclei contribute proportionately to the total vibrational spectrum of the material within the studied frequency range, the results correspond to the vibrational density of states in a thermodynamic context.

In general, the observed scattering *S*_obs_(*q*,ω) is a convolution of the scattering originating from the sample and the resolution function of the spectrometer. Given that the Boson peak is a broad feature in the scattering pattern, the convolution can be approximated by a summation. Under this assumption, eq 4 simplifies to^52^

$$S_{\mathrm{obs}}(q,\omega )=S_{\mathrm{inc}}(q,\omega )⊗R(q,\omega )\approx e^{-2W(q)}(R(q,\omega )+\frac{\hbar q^{2}}{2\mathrm{m̅}}\frac{g(\omega )}{\omega }\times (\exp(\frac{\hbar \omega }{k_{B}T})-1{)}^{-1})$$ 5

where *R*(*q*,ω) is the resolution function of the spectrometer. Equation 5 represents a linear relationship for the density of states and *R*(*q*,ω). By measuring the scattering at two different temperatures (here 2 and 50 K), the functions *g*(ω) and *R*(ω) can be estimated. Fundamentally, the density of states should be independent of the scattering vector under the incoherent approximation. In practice, eq 4 yields values decreasing with *q* due to multiple scattering contributions at low *q*. To enhance accuracy, *g*(ω) is extrapolated from the detectors within the angular range of 53–124°, corresponding to an elastic *q* range of 1.1–2.4 Å^–1^. Because of the factor *q* ^2^ in eq 5, incorporating smaller angles would not result in a significant statistical improvement.

Figure 3a compares *g*(ω)/ω^2^ versus angular frequency for P1MA and poly­(methyl phenyl siloxane) (PMPS). The data for the latter polymer were taken from ref 25. A comparison of PnMAs and PMPS is particularly informative because both polymer types have flexible backbones and PMPS does not exhibit the nanophase separation like P1MA. Given that PMPS is a reference material with an established Boson peak, this comparison enables the isolation of nanophase separation effects on the VDOS. In consequence, a similar BP behavior is expected for the two materials, as it is primarily influenced by the amorphous structure and the intrinsic flexibility of the backbone. As a first result, as expected, P1MA shows a Boson peak at a frequency of about 2.6 ps^–1^. This frequency range is similar to that of the BP for PMPS. The increase in *g*(ω)/ω^2^ at low frequencies is probably due to quasielastic contributions from the methyl group rotation because not all modes of this process are completely frozen at 50 K.^34^ This interpretation is in agreement with the observation that these contributions decrease with increasing alkyl side chain length, as with increasing *n*, the relative contribution of the methyl groups to the signal decreases (see Figure 4). Apart from a smaller intensity of the BP of P1MA, the Boson peaks of PMPS and P1MA are quite similar with regard to their position as discussed above and shape. This becomes clearer in Figure 3b where *g*(ω)/ω^2^ is divided by its maximum value and plotted versus frequency reduced by the maximum frequency of the Boson peak. In this representation, the low-frequency vibrational density of states of both polymers collapses nearly into one master curve.

**Figure 3:**
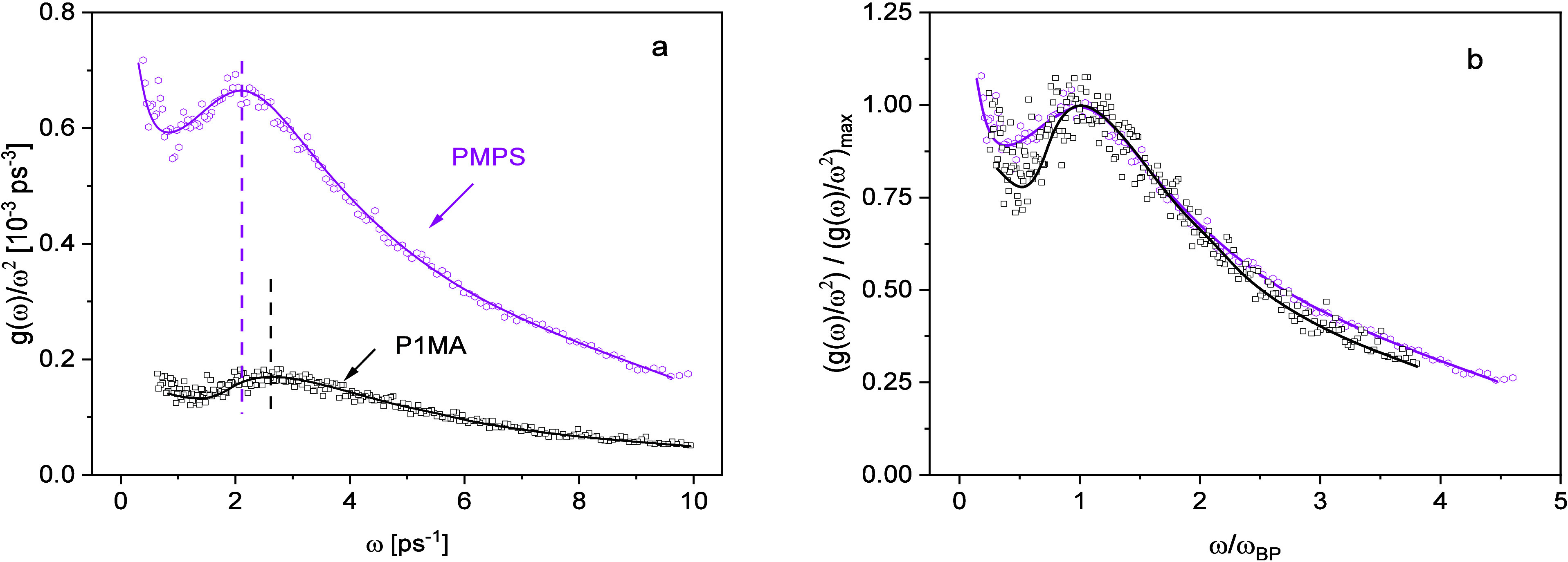
(a) Comparison of the vibrational density of states normalized by ω^2^ vs frequency for P1MA (black squares) and PMPS (pink hexagons). The data for PMPS are taken from ref 25. Lines are guides to the eyes. The increase in *g*(ω)/ω^2^ for low frequencies is due to quasielastic contributions because not all nonvibrational molecular motions are completely frozen at 50 K.^34^ (b) *g*(ω)/ω^2^ divided by its maximum value versus frequency reduced by the maximum frequency of the Boson peak ω_BP._ Black squares: P1MA; pink hexagons: PMPS. Lines are guides to the eyes.

**Figure 4:**
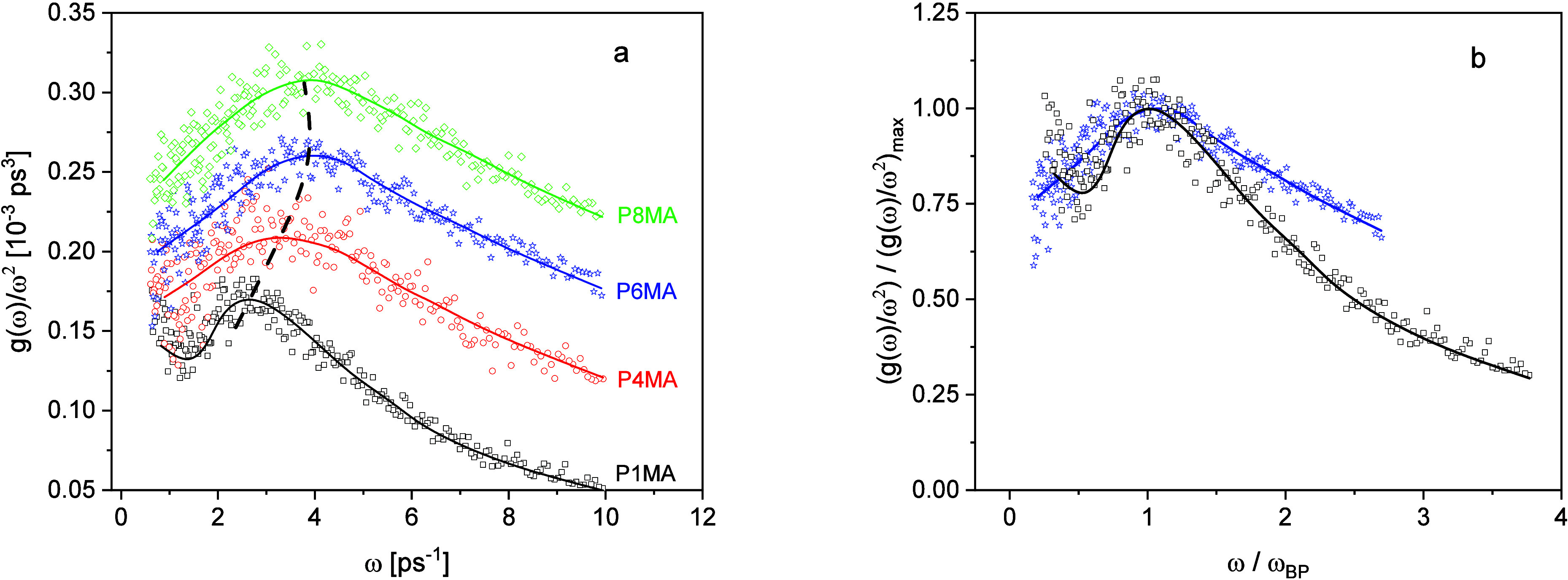
(a) Comparison of the vibrational density of states normalized by ω^2^ vs frequency for the PnMA as indicated. The curves are shifted along the *y*-scale for the sake of clarity: P4MA + 0.05, P6MA + 0.12, and P8MA + 0.17. Lines are guides for the eyes. The dashed line indicates the shift of the maximum frequency of the Boson peak as guides to the eyes. (b) *g*(ω)/ω^2^ divided by its maximum value versus frequency reduced by the maximum frequency of the Boson peak ω_BP_. Black squares: P1MA; blue asterisk: P6MA. Lines are guides to the eyes.

Figure 4a compares the Boson peak of P1MA with those of the other members of the homologous series. The BP for PnMAs with *n* > 1 is essentially broader than that of P1MA. This is demonstrated by Figure 4b with the same scaling, where *g*(ω)/ω^2^ is reduced by (*g*(ω)/ω^2^)_max_ and plotted versus frequency reduced by the maximum frequency of the BP. It is worth mentioning that the broadening appears at the low- and high-frequency side of the BP. The scaling, which is observed for P1MA and PMPS, is not fulfilled for the homologous series of poly­(*n*-alkyl methacrylate)­s for *n* > 1. It might be argued that the change of the shape of the Boson peak for *n* > 1 might be due to the nanophase separation.

The spectral shape of the Boson peak is quantitatively analyzed by fitting an empirical formula based on theoretical considerations to the data. The fit function given in ref 53 is expressed in *g*(ω)/ω^2^ versus ω by

$$\frac{g(\omega )}{\omega^{2}}=\frac{3}{\omega_{0}^{2}}\sqrt{\frac{a+\omega^{2}}{b+\omega^{4}}}$$ 6

In this model for low frequencies, *g*(ω) approaches the sound wave limit 3ω^2^/ω_D_^3^ where ω_D_ is the Debye frequency in the sound wave approach. For high frequency, eq 6 results in the density of states resulting from the eigenvalue spectrum of a random matrix. For more details, see ref 53. In eq 6, ω_0_ is a scaling parameter. The fitting parameters *a* and *b* have no distinct physical meaning, but they are related to ω_D_ and the maximum frequency of the BP ω_BP_. Figure S2 in the Supporting Information shows that the experimental data are well-described by this approach. The relationship of the fit parameter to ω_BP_ is given by

$$\omega_{BP}=\sqrt{\sqrt{a^{2}+b}-a}$$ 7

while the Debye level of the sound wave approach is calculated using

$$D=\lim_{\omega \to 0}\frac{g(\omega )}{\omega^{2}}=\frac{3}{\omega_{0}^{2}}\sqrt{\frac{a}{b}}$$ 8

The dependence of ω_BP_ on the number of the carbon atoms in the side chain is depicted in Figure 5a. With increasing length of the alkyl side chain, the maximum frequency of the BP shifts to higher values and seems to reach a maximum for *n* = 8 and might be attributed to a change in the characteristic length scale ξ and/or the transverse sound velocity.

**Figure 5:**
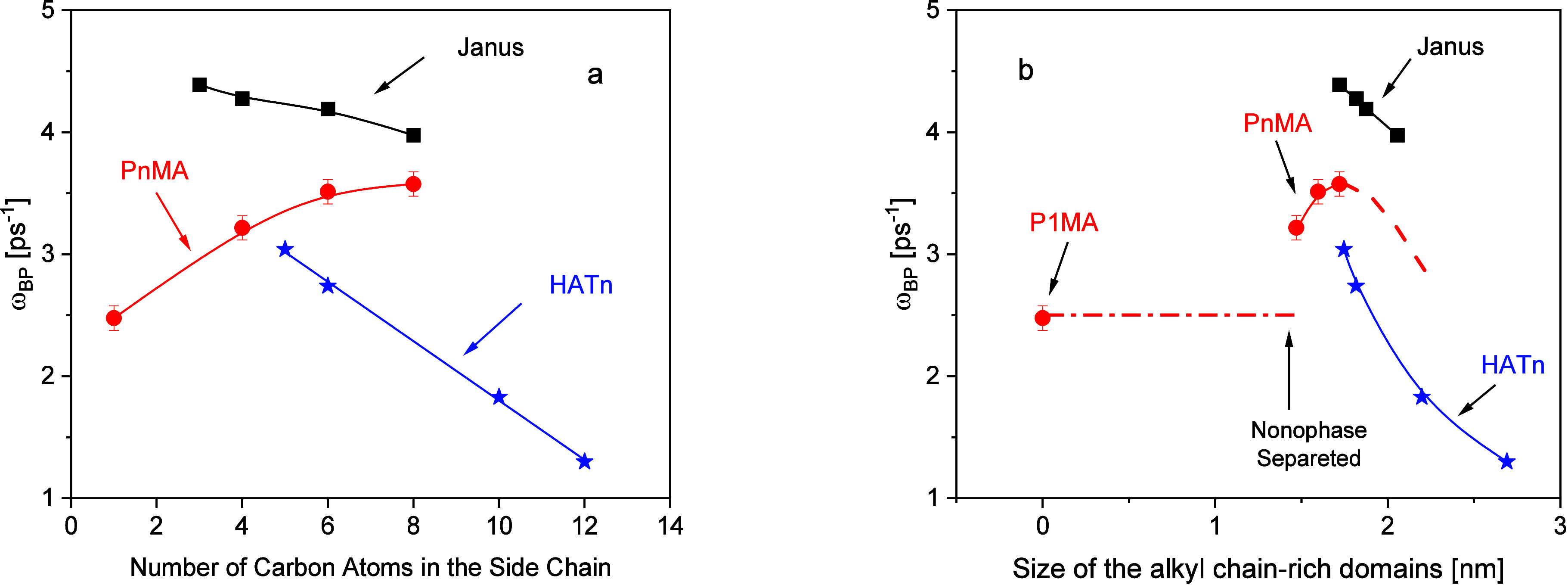
(a) Maximum frequency of the Boson peak ω_BP_ versus the number of carbon atoms in the side chain for different nanophase-separated systems having alkyl side chains: solid red circles, PnMA; black solid squares, Janus-polynorbornenes; the data were taken from ref 36. Blue solid asterisk: discotic liquid crystal HATn; the data were taken from ref 10. Lines are guides for the eyes. The errors are estimated from the fit. (b) Maximum frequency of the Boson peak ω_BP_ versus size of the alkyl side chain-rich domains. The color code is the same as in part (a) of the figure. Lines are guides for the eyes. The dashed red line is the expected behavior for *n* > 8.

For the discussion of the dependence of the maximum frequency of the Boson peak ω_BP_, eq 2 is employed. Besides *c_t_*, eq 2 contains a characteristic length scale ξ. Notably, a characteristic length exists also for P1MA as a Boson peak is observed also for this material. Unfortunately, the characteristic length scale ξ is not known for the PnMAs. However, for *n* > 1, the domain size of the alkylside chain-rich domains might be considered as the dominating length scale. Using this length scale according to eq 2, the calculated transverse sound velocity would increase with increasing alkyl side chain length (see the Supporting Information, Figure S3). This seems unlikely, as the elastic modulus below the glass transition temperature decreases with increasing side chain length,^54,55^ which should, in turn, lead to a decrease in the transverse sound velocity, *c_t_*. Moreover, as discussed below, the Debye level drops for *n* > 4 (see Figure 6b). Since the Debye level is inversely related to the transverse sound velocity, this dropdown would mean an increase in *c_t_* contradicting the observed decrease in the elastic modulus. This discrepancy suggests that additional factors, such as nanophase separation, may be influencing the behavior beyond just the sound velocity. It is worth noting that, in the context of this discussion, experimental values of the transverse sound velocity, particularly at 50 K, would be highly beneficial. However, a thorough review of the literature indicates that such data seem to be currently unavailable. Moreover, the behavior observed for the PnMAs is different from that obtained for comparable systems, which undergo also a nanophase separation with a similar size of the alkyl side chain-rich domains (see Figure S1 and discussion below). Therefore, it might be again concluded that additional effects must be taken in account.

**Figure 6:**
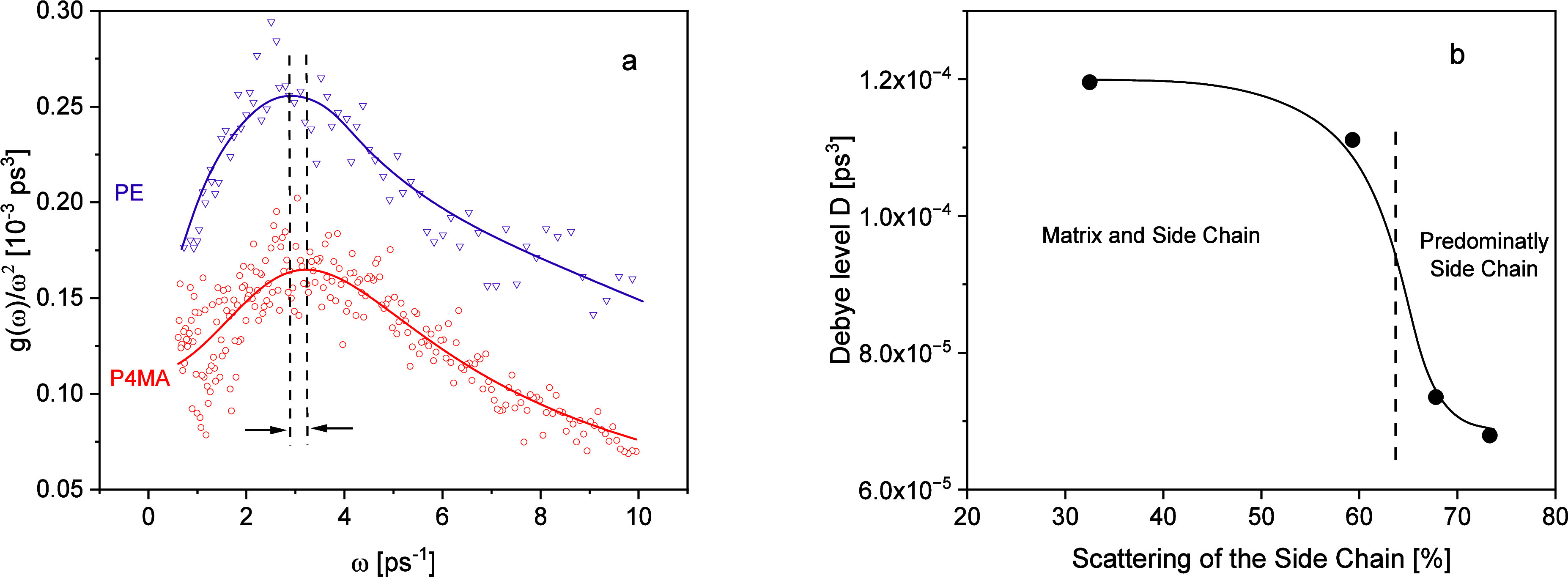
(a) *g*(ω)/ω^2^ for P4MA (red circles) and PE (violet down triangles). The data for PE were taken from ref 56. Please note that no absolute units were provided for PE in ref 54. Lines are guides to the eyes. Therefore, the data were arbitrarily scaled. Lines are guides for the eyes. (b) Debye level *D* versus the calculated scattering contribution coming from the side chain. The line is a guide for the eyes.

Figure 5a shows data of the maximum frequency of the Boson peak versus the number of the carbon atoms for systems having long alkyl side chains and undergoing nanophase separation. These data include values for semirigid Janus-polynorbornenes^39^ as well as for the discotic liquid crystal system hexakis­(*n*-alkyloxy)­triphenylene (HATn).^10^ The structures of the Janus-polynorbornenes and HATn are given in the Supporting Information (see Figure S4). In contrast to PnMA, for the Janus-polynorbornenes and the HATn, the maximum frequency of the Boson peak decreases with increasing length of the alkyl side chain. This behavior agrees with eq 2 as the materials become softer with increasing side chain length. It seems that the data for the PnMA approach the dependency of the Janus-polynorbornenes for *n* = 8. Of course, the molecular structures of the considered materials are highly different. PnMAs have a relative flexible backbone with one alkyl side chain per repeating unit. The Janus-polynorbornenes are characterized by a semirigid backbone with three alkyl side chains per repeating unit. The HNTn consists of low-molecular-weight disk-like molecules with a rigid core and six alkyl side chains per molecule. However, the higher-level morphology of all these materials is similar to that of a nanophase-separated system of a matrix with incorporated alkyl side chain-rich domains. This means that a similar dependence of the maximum frequency of the Boson peak on the length of the alkyl side chain should be expected. Why the dependence of the maximum frequency of the Boson peak of the PnMAs is different compared to that of the Janus-polynorbornenes and HATn will be discussed in detail below.

As the PnMAs undergo a microphase separation for *n* > 1 (see ref 40), like for the Janus-polynorbornenes and the HATn, the maximum frequency of the Boson peak can be also plotted versus the domain size (see Figure 5b). It is worth mentioning that no theoretical assumption or model is used to draw Figure 5b. First, in this representation, the maximum frequency of the Boson peak is higher for P4MA compared to that of P1MA, which represents mainly the BP of the matrix. To discuss this difference, the BP of P4MA is matched to that of “amorphous” polyethylene (PE) in Figure 6a. Here, “amorphous” PE means that the contribution of the amorphous parts of polyethylene segments constrained or confined between crystalline lamellae is measured. This figure shows that the position of the BP for P4MA compares to that of PE. Therefore, it is concluded that the shifted position of the BP of the PnMA for *n* > 1 in comparison to that of P1MA is also due to the methylene units confined in the alkyl side chain-rich domains and therefore to confined vibrations. In other words, for P4MA, the BP peak is due to a dominant response of the alkyl side chain confined in the alkyl side chain-rich domains. Second, as the domain size increases with increasing length of the alkyl side chain, ω_BP_ shifts to higher frequency with increasing domain size reaching a maximum (Figure[Fig fig5]b). Nevertheless, in the representation ω_BP_ versus domain size, the maximum of ω_BP_ is more pronounced. From that argument, it can be expected that for longer alkyl side chain lengths than *n* = 8, ω_BP_ decreases like for the Janus-polynorbornenes and HATn. In further works, this hypothesis will be further proven considering PnMAs with side chain length *n* > 8 and/or deuterating its backbones.

The decrease in the ω_BP_ for the Janus-polynorbornenes and HATn was discussed by a continues release of the confinement effect on the phonons with increasing size of the alkyl side chain-rich domains, thus with increasing *n*. At this point, the question appears what is the reason why for the Janus-polynorbornenes and HATn, the frequency of the Boson peak decreases continuously with increasing size of the alkyl side chain-rich domains while for PnMA ω_BP_, it first increases going probably through a maximum and is expected to decrease when the size of the alkyl side chain-rich domains increases further (see Figure 5b). The Janus-polynorbornenes have three alkyl side chain per monomeric unit. Furthermore, HATn has six alkyl side chains per molecule. This means that the contribution of the backbones of the Janus-polynorbornenes or the cores of HATn to the whole scattering is low. This is different for PnMA as the backbone contains five hydrogen nuclei (two in the main chain and three in the methyl group, see Figure 1). For the nanophase-separated P4MA, 59.3% of the calculated incoherent scattering is due to the alkyl side chains (see Table 2). This means that 40.7% of the incoherent scattering comes from the matrix. Therefore, the dependence of the maximum frequency of the Boson peak can be considered as a counterbalance of the scattering of the matrix and that of alkyl side chain-rich domains. This becomes also clear considering the Debye level of the sound wave approach to the BP versus the calculated scattering contribution of the alkyl side chains (see Figure 6b). The Debye level was obtained from the fit of eq 6 to the Boson peak and calculated from the fit parameters by eq 8. The Debye level decreases slightly from P1MA to P4MA followed by a-step-like decrease reaching a kind of plateau. Notably, the strong change of the Debye level with increasing scattering from the side chain for *n* > 4 is not an artifact of the fit, it is visible in the raw data itself (Supporting Information, Figure S5). For a scattering arising only from the alkyl side chain-rich domains, a constant value of *D* should be expected. Therefore, the change in the Debye level with increasing alkyl side chain length indicates a change in the scattering from the matrix to alkyl side chain-rich domains.

The anomalies in the temperature dependence of the specific heat capacity *c_p_* due to excess contributions of the low-frequency vibrational density of states for the considered homologous series of the poly­(*n*-alkyl methacrylate)­s were studied in ref 2. In the representation *c_p_*/*T* ^3^ versus temperature, a maximum was revealed, where the maximum temperature $T_{\max,c_{p}}$ shifts to higher values with increasing length of the alkyl side chain. In Figure 7a, $T_{\max,c_{p}}$ taken from ref 2 is plotted versus ω_BP_ according to eq 1 (black circles). The figure shows that $T_{\max,c_{p}}$ scales almost linearly with the maximum frequency of the Boson peak. Nevertheless, a free linear regression will not go to the point of origin as expected (black line in Figure 7a). This small discrepancy might be because the spectral shape of the BP is changed from P1MA to P4MA (see Figure 4a). However, forcing the fit to go through the point of origin will lead to a description of the data with a comparable quality (see the red line in Figure 7a). See also the consideration given in the SI.

**Figure 7:**
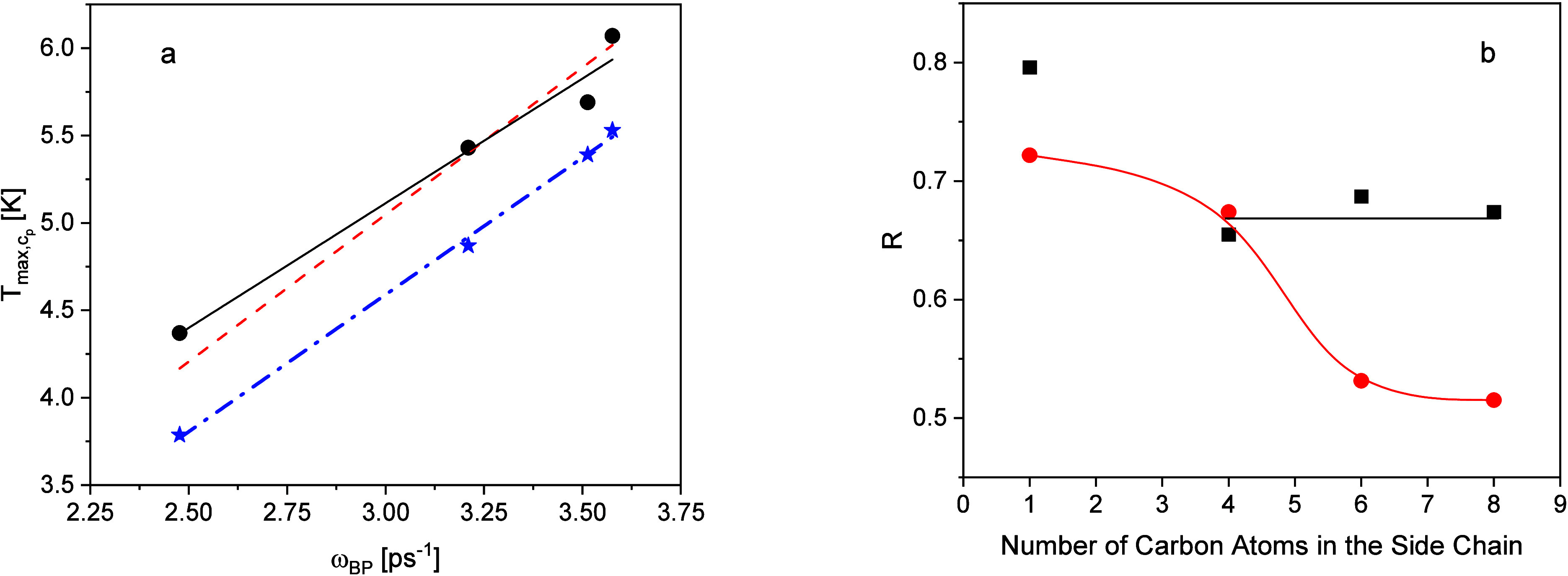
(a) Maximum temperature of *c_p_*/*T* ^3^ versus maximum frequency of the BP measured by neutron scattering. The black circles correspond to experimental results taken from from ref 2. The black solid line is a free linear regression to the data. The red dashed line is a linear regression forced going through the point of origin. The blue asterisks are taken from the specific heat calculated from the low-frequency vibrational density of states (see eq 11 and Figure 8). The dash-dotted line is a linear regression. (b) Sokolov ratio *R* versus the number of carbon atoms in the side chain. Red circles: data from neutron scattering in this work. The line is a guide for the eyes. Black squares: thermal data taken from ref 2.

To characterize the Boson peak in more detail, Sokolov et al. defined a ratio *R* ^57,58^ between minimum and maximum of the reduced VDOS by

$$R=\frac{{\left(\frac{g(\omega )}{\omega^{2}}\right)}_{\min}}{{\left(\frac{g(\omega )}{\omega^{2}}\right)}_{\max}}<1$$ 9

is considered. It is worth noting that ${\left(\frac{g(\omega )}{\omega^{2}}\right)}_{\min}$ is the Debye level *D*. In this notation, *R* = 1 means that no BP is observed, which corresponds to a perfect crystal structure. In refs 55 and 56, it was shown that *R* could be related to the fragility. This relationship between *R* and a dynamic fragility is found to be valid for the considered series of poly­(*n*-alkyl methacrylate)­s.^2^ *R* can be calculated from the fit parameters of the BP by

$$R=\sqrt{\frac{a(b+(\sqrt{a^{2}+b}-a{)}^{2})}{b\sqrt{a^{2}+b}}}$$ 10

and is plotted in Figure 7b versus the number of the carbon atoms in the side chain.

With an increasing number of carbon atoms, *R* decreases reaching a kind of plateau for the highest values of *n*. The ratio *R* can be also estimated from the heat capacity in the representation *c_p_*/*T* ^3^.^2^ The data given in ref 2 for the considered homologous series of the poly­(*n*-alkyl methacrylate)­s are included in Figure 7b. Also, *R* deduced from the thermal data decreases with an increasing number of the carbon atoms in alkyl side chains and reaching a plateau for the highest values of *n*. Nevertheless, the *R* values estimated from the thermal data are higher than those from the Boson peak measured by neutron scattering. It could be shown by model calculations using eq 6 that the values of *R* estimated from the specific heat are always higher than those obtained from *g*(ω) (see the SI). Roughly speaking, this is due to the fact that the peak in *c_p_*/*T* ^3^ is always shallower than that of *g*(ω)/ω.^2^ In that respect, see also eq 11.

The temperature dependence of the specific heat capacity *c_V_* can be calculated from the vibrational density of states by^59^

$$c_{V}=\frac{3k_{B}}{\mathrm{m̅}}\int_{0}^{\omega_{\max}}{\left(\frac{\hbar \omega }{k_{B}T}\right)}^{2}\frac{e^{\hbar \omega /k_{B}T}}{(e^{\hbar \omega /k_{B}T}-1{)}^{2}}g\left(\omega \right)d\omega$$ 11

From the theoretical point, the integration of eq 11 should be carried out until the frequency ω_D_ given by $\int_{0}^{\omega_{D}}g(\omega )d\omega =1$ , which is the definition of Debye frequency for the Debye model. However, the time-of-flight spectrometer provides only a limited frequency window where the Debye frequency is higher than the highest accessible frequency by FOCUS. Therefore, for the estimation of maximal frequency ω_max_ for the integration of eq 11, *g*(ω) was extrapolated from its value at the highest frequency reliably accessible by the FOCUS instrument until $\int_{0}^{\omega_{\max}}g\left(\omega \right)d\omega =1$ is reached. First, this means that ω_max_ is not the Debye frequency. Second, the extrapolation of *g*(ω) over a larger frequency range leads also to a higher uncertainty in the values of ω_max_. This calculation leads for P1MA to ω_max_ = 173 ps^–1^. The calculated values of ω_max_ for all PnMA materials are given in the SI (see Table S2). In principle, the Debye level and the Debye frequency should be related by *D* ∼ ω_D_^–3^. Such a correlation is not observed for *D* and ω_max_ (see Figure 6b and Table S2). The possible reasons are that ω_max_ represents not the value of ω_D_ and that ω_max_ is subjected to higher errors. To avoid an extrapolation of *g*(ω) at low frequencies, the Debye level obtained from the fit of eq 6 to the Boson peak was used for the calculation of the specific heat. Furthermore, eq 11 gives the specific heat capacity at constant volume where the experimental data were measured at constant pressure (*c_p_*).^2^ *c_p_* is always a bit greater than *c_V_* depending on temperature, but it is known that the difference is small. This is especially true for low temperatures.^57^ Therefore, for the following discussion, *c*_*p*_ ≈ *c_V_* was assumed. Moreover, for a comparison with experimental data, it is worth noting that eq 11 covers only vibrational contributions to the specific heat. Tunneling contributions are not considered by that equation. The specific heat capacity was calculated from the low-frequency vibrational density of states measured by neutron scattering assuming that *g*(ω) reaches the Debye level at low frequencies. This means that *g*(ω) is a constant at low frequencies leading to *c_V_* (*T*)/*T* ^3^ = const for low temperatures. The specific heat capacity calculated from the vibrational density is compared to the experimental values given in ref 2 in Figure 8.

**Figure 8:**
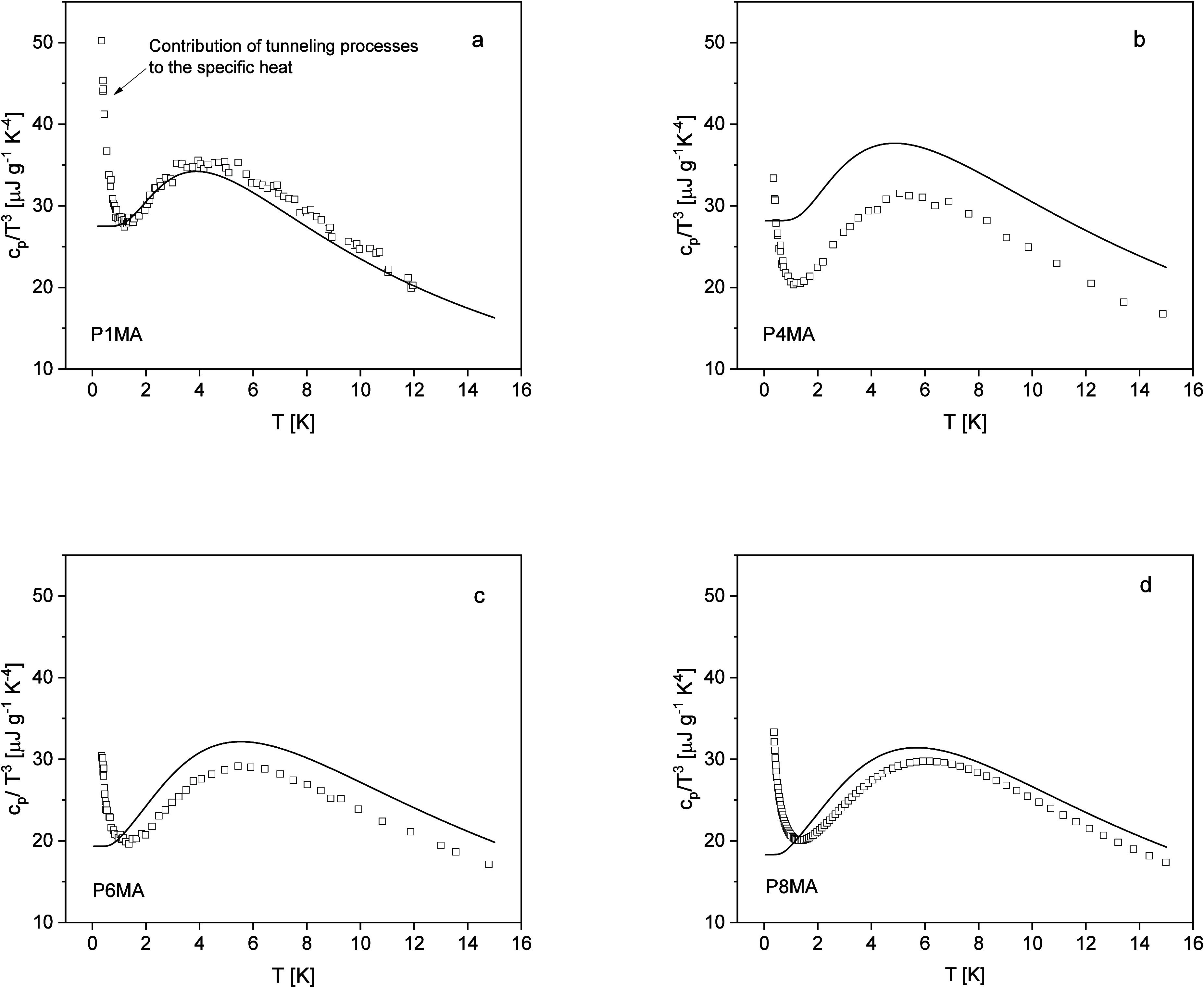
*c_p_*/*T* ^3^ versus temperature. Open squares: experimental values taken from ref 2; solid lines: calculated according to eq 11. (a) P1MA, (b) P4MA, (c) P6MA, and (d) P8MA.

The comparison of the calculated values with the experimental ones reveals that the temperature dependence of the specific heat calculated from the low-frequency vibrational density of states is in qualitative agreement with the experimental values. This agreement concerns approximately the absolute values of the specific heat, the temperature position of the Boson peak, and its shape. Nevertheless, also differences between the experimental data and the calculated ones are observed. For a quantitative comparison of the calculated values with the experimental ones, one must keep in mind that the equation to estimate the low-frequency vibrational density from the incoherent dynamic scattering function (eq 4) was derived for one kind of atom arranged on a cubic lattice.^51^ For the amorphous system considered here, which contains also different nuclei, the situation is more complex. To justify the calculation of the specific heat capacity from the estimated low-frequency density of states (see eq 11), one can argue that (a) the vibration arises from acoustical phonons. This means that the mean-squared displacement due to the hydrogen nuclei is like the average. If this assumption is not completely fulfilled, then one would expect an overestimation of *g*(ω).^51^ (b) A cubic lattice is the closest situation to an amorphous structure, which is also isotropic in all three dimensions.

The calculated temperature positions of *c_V_*/*T* ^3^ are a bit lower than the experimental ones. To quantify this difference, $T_{\max,c_{p}}$ calculated from the low-frequency vibrational density of states is included in Figure 7a. This comparison shows that the difference between the experimental values is around 0.5 K for *n* = 1 and 4 and even smaller for *n* = 6 and 8. The calculated data follow a straight line, which goes closely to the point of origin in an unbiased linear regression.

The absolute values of the calculated values of *c_p_*/*T* ^3^ agree best with the data of P1MA. For *n* > 1, the calculated values are a bit higher than the experimental ones. The largest deviation between the calculated and experimental values are found for P4MA where the calculated values are by a factor of 1.2 higher than the experimental ones. In ref 60, it was argued that the specific heat capacity calculated from *g*(ω) could be larger by a factor of 3.3 due to the contributions of different nuclei with a similar amplitude of the vibration to the scattering. This factor is related to differences in the scattering cross sections and the masses of the atoms. In the case considered here, the largest factor is found to be 1.2 although the mass ratios are larger than in the case of ref 57. This means that the observed differences can be considered as small.

## 4 Conclusions

This study provides a comprehensive estimation and analysis of the low-frequency vibrational density of states (VDOS) in nanophase-separated poly­(*n*-alkyl methacrylate)­s (PnMAs) and their relationship to the specific heat capacity. Using inelastic neutron scattering, the low-frequency vibrational density of states was calculated from the incoherent scattering function in the one-phonon approximation. It was observed that the Boson peak (BP) shifts to higher frequencies for the nanophase-separated PnMAs (*n* > 1) with increasing alkyl side chain length reaching a maximum. After the maximum, it is expected that the maximum frequency will decrease with further increasing the length of the alkyl side chain. These results indicate a counterbalance between confinement effects and the scattering resulting from the matrix. This counterbalance was also evidenced by a step-like change of the Debye level on calculated scattering of the alkyl side chains. From these results, evidence is concluded that the Boson peak in PnMA is due to collective effects like sound waves particularly confined in the alkyl side chain-rich domains. With increasing size of the alkyl side chain-rich domains, confinement effects are released. This behavior was compared to other nanophase-separated systems, such as Janus-polynorbornenes and HATn discotic liquid crystals.

The study confirms an approximal linear relationship between the maximum frequency of the BP and the maximum temperature of the specific heat anomaly. From the measured low-frequency vibrational density, the temperature dependence of the specific heat capacity is calculated. The calculated specific heat capacity from the VDOS shows qualitative agreement with experimental data.

The obtained results demonstrate that the nanophase separation in PnMAs significantly influences their vibrational properties. The confinement of phonons within the alkyl side chain-rich domains leads to distinct shifts in the BP, which are indicative of the interplay between the polymer matrix and the nanophase-separated domains. This interplay is crucial for understanding the thermal and mechanical properties of these materials.

Furthermore, the comparison with other nanophase-separated systems, such as Janus-polynorbornenes and HATn discotic liquid crystals, highlights the universality of the Boson peak also for nanophase-separated materials. Despite differences in chemical structure and morphology, these systems might exhibit similar trends in the behavior of the BP, suggesting that the underlying mechanisms are broadly applicable across different types of nanophase-separated polymers. This conclusion will be further proven considering PnMAs where the main chain is deuterated.

The study also underscores the importance of considering both the molecular structure and the mesoscale morphology when analyzing the vibrational properties of polymers. The observed shifts in the BP with varying alkyl side chain lengths provide insights into the design of polymers with tailored thermal and mechanical properties. By controlling the length of the alkyl side chains, it is possible to fine-tune the vibrational properties and, consequently, the specific heat capacity of the material at low temperatures.

In conclusion, this research enhances our understanding of the molecular dynamics and phase behavior of PnMAs, contributing valuable insights into the vibrational properties of nanophase-separated polymers. Future work could explore the effects of different confinement environments and polymer architectures on the BP and related thermodynamic properties. Additionally, further studies could investigate the impact of external factors, such as temperature and pressure, on the VDOS and BP in these materials. Such investigations would provide a more comprehensive understanding of the factors influencing the vibrational properties of nanophase-separated polymers.

## Supplementary Material



## Notes

Size of the alkyl chain-rich domains versus the number of carbon atoms in the side chains for PnMA, Janus-polynorbornenes, and HATn; calculation of the scattering cross sections of the repeating units for the different polymers; analysis of the spectral shape of the Boson peak; chemical structure of the Janus-polynorbornenes and HATn; values of the Debye frequencies for the PnMA; relation between the Boson peak in *g*(ω) and *c_V_* (*T*)/*T* ^3^ (PDF)
