# Supplementary material for: Low-Frequency Vibrational Density of States of Nanophase-Separated Poly(n‑alkyl methacrylate)s: Confined Phonons and Relationship to Specific Heat
Source: Macromolecules. 2025 Jul 9;58(14):7112–23. doi: 10.1021/acs.macromol.5c00898 (PMC12288068; doi:10.1021/acs.macromol.5c00898)
Supplement: Supplementary file 1 [file ma5c00898_si_001.pdf]

Supporting Information

Low frequency vibrational density of states of nanophase separated poly(n-alkyl methacrylate)s – Confined phonons and relationship to the specific heat

Paulina Szymoniak[1], Fanni Juranyi[2], Margarita Kruteva[3], Reiner Zorn[3] and Andreas Schönhals[1,4,*]

[1]Bundesanstalt für Materialforschung und -prüfung (BAM), Unter den Eichen 87, 12205 Berlin, Germany

[2]Paul Scherrer Institut (PSI), Forschungsstraße 111, 5232 Villigen, Switzerland

[3]Forschungszentrum Jülich GmbH, Jülich Centre for Neutron Science (JCNS-1), 52425 Jülich, Germany

[4]Institut für Chemie, Technische Universität Berlin, Straße des 17. Juni 135, 10623 Berlin, Germany

[*]CORRESPONDING AUTHOR: A. Schönhals, BAM Bundesanstalt für Materialforschung und -prüfung (Department Materials Chemistry), Unter den Eichen 87, 12205 Berlin, Germany; Tel. +49 30 / 8104-3384; Fax: +49 30 / 8104-73384; Email: Andreas.Schoenhals@bam.de

Dependence of size of the alkyl side chain-rich domains on the number of the carbon atoms in the side chain.

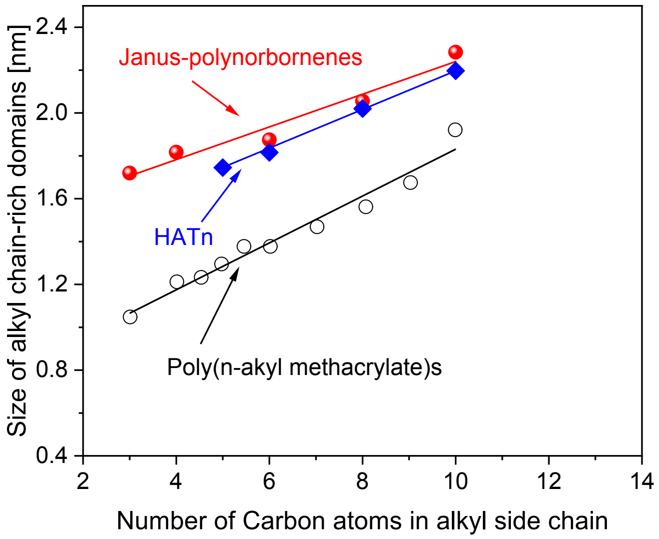

Figure S1: Dependence of size of the akyl side chain-rich domains on the number of the carbon atoms in the side chain as indicated. The data for the poly(n-alkyl methacrylate)s were taken from ref. S1. The data for HATn were taken from ref. S2, The data for the Janus-polynorbornenes were taken from ref. S3.

Calculation of the scattering cross sections

Calculation of the neutron cross sections for the materials used. The total cross sections were obtained by multiplication of the individual nuclear cross sections with the number of respective nuclei in the monomeric units. This procedure is correct for incoherent scattering and absorption; for coherent scattering it represents the high-q limit. The absorption cross sections were corrected to the actual wavelength of the experiments, and therefore the tabulated values are multiplied a factor 5 Å / 1.8 Å in addition.

Table S1: Neutron scattering cross-sections.

P1MA

Number	Atom	coh. ind.	coh. total	inc. ind.	inc. total	abs. ind.	abs. total
5	C	5.551	27.755	0.001	0.005	0.0035	0.049
9	H	1.7568	15.811	80.26	722.34	0.3326	8.381
2	O	4.232	8.464	0.0008	0.0016	0.00019	0.00106
Total / monomeric unit:			52.03		722.346		8.431

P4MA

Number	Atom	coh. ind.	coh. total	inc. ind.	inc. total	abs. ind.	abs. total
8	C	5.551	44.408	0.001	0.008	0.0035	0.0784
15	H	1.7568	26.352	80.26	1203.9	0.3326	13.97

2	O	4.232	8.464	0.0008	0.0016	0.00019	0.00106
Total / monomeric unit:			79.224		1203.909		14.05

P6MA

Number	Atom	coh. ind.	coh. total	inc. ind.	inc. total	abs. ind.	abs. total
10	C	5.551	55.51	0.001	0.01	0.0035	0.098
19	H	1.7568	33.38	80.26	1524.95	0.3326	17.694
2	O	4.232	8.464	0.0008	0.0016	0.00019	0.00106
Total / monomeric unit:			97.353		1524.951		17.793

P8MA

Number	Atom	coh. ind.	coh. total	inc. ind.	inc. total	abs. ind.	abs. total

12	C	5.551	66.61	0.001	0.012	0.0035	0.1176
23	H	1.7568	40.406	80.26	1845.98	0.3326	21.419
2	O	4.232	8.464	0.0008	0.0016	0.00019	0.00106
Total / monomeric unit:			115.48		1845.99		21.54

Analysis of the Boson peak with the semiempirical fit function given in eq. 5.

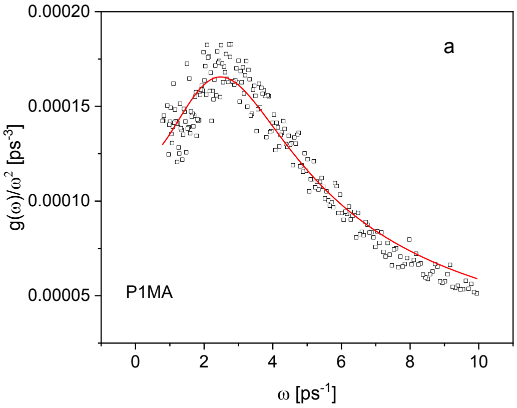

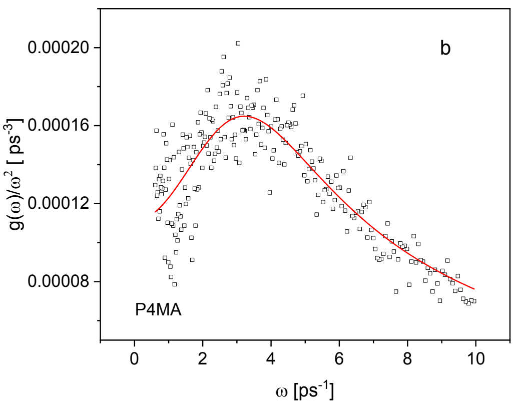

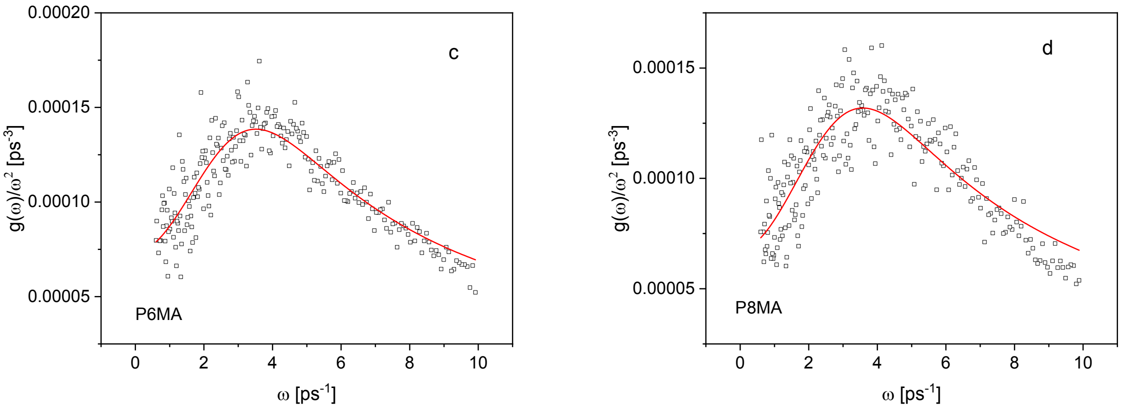

Figure S2: Analysis of the Boson peak by fitting eq. 5 to the data. Open squares – experimental data, red line – fit. a – P1MA; b – P4MA; c – P6MA; d – P8MA

Approximation for the transverse sound velocity

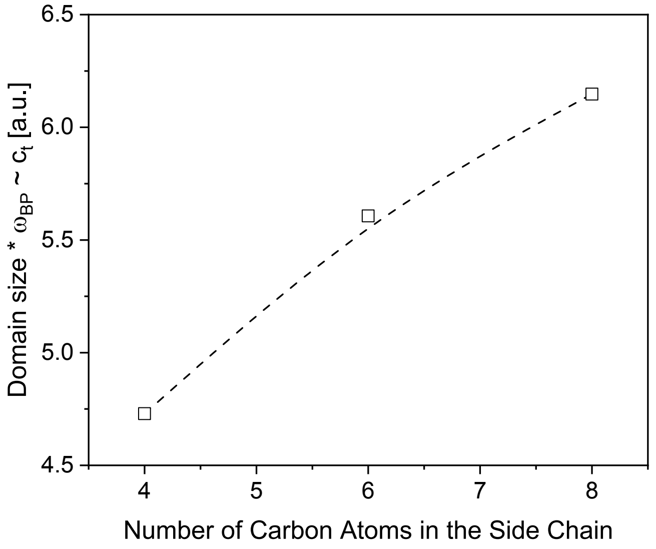

Figure S3: Domain size×ω_{BP} as approximation for the transverse sound velocity versus the number of carbon atoms in the side chain for n>1. The line is a guide for the eyes.

Chemical structure of the Janus - polynorbornenes and HATn.

a

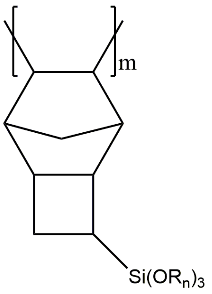

R symbolizes the alkyl side chain with 3, 4, 6 and 8 carbon atoms

b

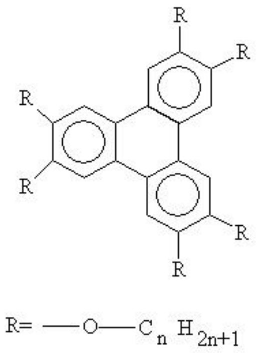

The number of carbon atoms in the side chain n has values n=5,6,10,12.

Figure S4: Chemical structure of the Janus-polynorbornenes (a) and HATn (b).

Table S2: Values of the maximum frequency used for the calculation of c_V from the low frequency vibrational density of states.

Code	PAMA	P4MA	P6MA	P8MA
ω_{max} [ps^{-1}]	164	119	163	174

Comparison Debye level

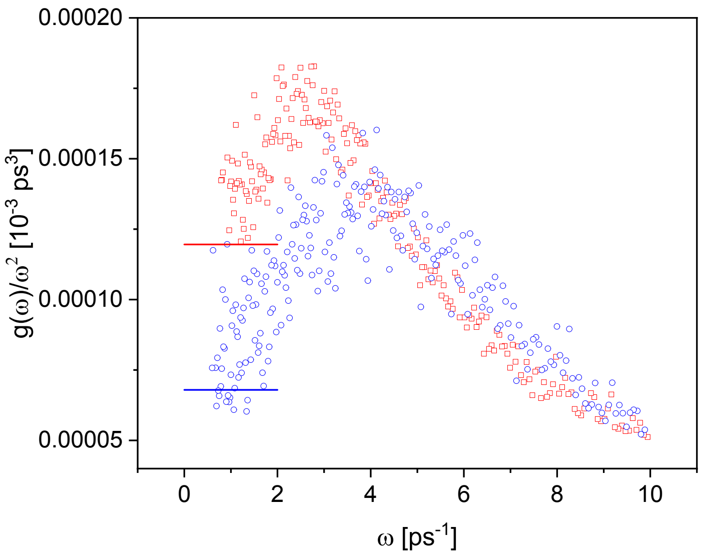

Figure S5: Comparison of the vibrational density of states normalized by ω^2 vs. frequency for P1MA (red squares) and P8MA (blue circles). Red line – obtained Debye by the fit of eq. 6 to the data for P1MA. Blue line – obtained Debye by the fit of eq. 6 to the data for P8MA.

Relation between the Boson peak in g(ω) and $c_V(T)/T^3$

For a precise comparison, one must note that the Sokolov R values calculated by low frequency vibrational density of states eq. 8 and those from the corresponding equation in terms of specific heat (see ref. S4)

$$R_{cV} = \left(\frac{c_V(T)}{T^3}\right)_{\min} \Big/ \left(\frac{c_V(T)}{T^3}\right)_{\max} \tag{S1}$$

do not exactly coincide. The reason is that, roughly speaking, equ. 10 'flattens' the peak. To analyze this more quantitatively, we have calculated the R values from both equations using the Buchenau model (equ. 5) for different combinations of a and b (in the limit of large ω_0 to avoid a cutoff from the upper limit of the integral in equ. 10). The results given in Figure S4 show that R_{cV} is systematically higher with a maximum deviation of about 0.075.

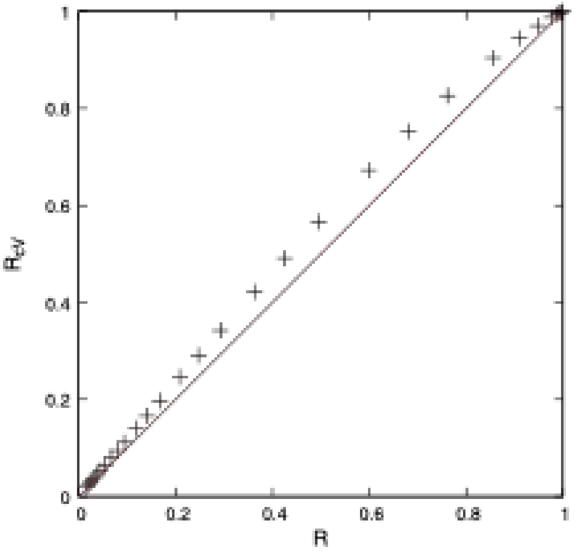

Figure S6: Comparison of R from equation (8) and R_{cV} from equation (S4) assuming the Buchenau model for the Boson peak (equ. 5). The values chosen for a were 0.001, 0.01…10 and for b 1, 2, 5, 10, 20, 50. The read line would represent R_{cV}=R.

In passing we note that also the peak positions are not directly comparable. For a delta-shaped peak (Einstein model), equ. 10 can be used for an exact relation: $k_B T_{\text{max, Specific heat}} = \hbar\omega_{BP}/A$, where A is the solution of the equation $A = 5\tanh(A/2)$, i.e. A=4.928… For a real Boson peak the factor 1/A depends on the shape. Assuming again the Buchenau model, the results in Figure S5 are obtained.

References:

S1 Beiner, M.; Huth, R. Nanophase separation and hindered glass transition in side-chain polymers. *Nature Materials* **2003**, 2, 595-59

S2 Krause, C.; Zorn, R.: Emmerling, F.; Falkenhagen, J.; Frick, B.; Huber, P.; Schönhals, A. Vibrational density of states of triphenylene based discotic liquid crystals: dependence on the length of the alkyl chain *Physical Chemistry Chemical Physics* **2014**, 16, 7324 – 7333.
S3 Kolmangadi, M.; Szymoniak, P.; Smales, G. J.; Alentiev, D.; Bermeshev, M.; Böhning, M.; Schönhals, A. Molecular dynamics of Janus polynorbornenes: Glass transitions and nanophase separation. *Macromolecules* **2020**, 53, 7410-7419.
S4 Beiner, M; Kahle, S.; Abens, S.; Hempel, E.; Höring, S.; Meissner, M.; Donth, E. Low-temperature heat capacity, glass-transition cooperativity, and glass-structure vault breakdown in a series of poly(n-alkyl methacrylate)s. *Macromolecules* **2001**, 34, 5927-5935.
